# Reduction of Methyltransferase-like 3-Mediated RNA N6-Methyladenosine Exacerbates the Development of Psoriasis Vulgaris in Imiquimod-Induced Psoriasis-like Mouse Model

**DOI:** 10.3390/ijms232012672

**Published:** 2022-10-21

**Authors:** Yanan Wang, Jiuzuo Huang, Hongzhong Jin

**Affiliations:** 1 Department of Dermatology, State Key Laboratory of Complex Severe and Rare Diseases, Peking Union Medical College Hospital, Chinese Academy of Medical Science and Peking Union Medical College, National Clinical Research Center for Dermatologic and Immunologic Diseases, Beijing 100730, China; 2 Department of Plastic Surgery, Peking Union Medical College Hospital, Chinese Academy of Medical Science and Peking Union Medical College, Beijing 100730, China

**Keywords:** METTL3, m^6^A methylation, N6-methyladenosine, psoriasis vulgaris, psoriasis, RNA modification

## Abstract

N6-methyladenosine (m^6^A) methylation is the most pervasive and intensively studied mRNA modification, which regulates gene expression in different physiological processes, such as cell proliferation, differentiation, and inflammation. Studies of aberrant m^6^A in human diseases such as cancer, obesity, infertility, neuronal disorders, immune diseases, and inflammation are rapidly evolving. However, the regulatory mechanism and physiological significance of m^6^A methylation in psoriasis vulgaris are still poorly understood. In this study, we found that m^6^A methylation and Methyltransferase-like 3 (METTL3) were both downregulated in psoriatic skin lesions and were negatively correlated with Psoriasis Area and Severity Index (PASI) scores. Inhibiting m^6^A methylation by knocking down *Mettl3* promoted the development of psoriasis and increased its severity in imiquimod-induced psoriasis-like model mice. Our results indicate a critical role of METTL3- mediated m^6^A methylation in the pathogenesis of psoriasis vulgaris.

## 1. Introduction

Psoriasis is a chronic, immune-mediated disease with an estimated global prevalence of 2% to 3%, which is often triggered by trauma, infection, and drugs in genetically susceptible populations [1]. Psoriasis vulgaris, as the most frequent phenotype, usually presents as well-defined, symmetrical, pink to erythematous, and scaly plaques on the scalp, trunk, and extremities [1]. The hallmark histopathological features of psoriasis vulgaris are epidermal acanthosis (thickening of viable layers), hyperkeratosis, and parakeratosis, accompanied by an inflammatory infiltrate in the dermis [2]. The complex interactions between keratinocytes and T cells are responsible for the development and maintenance of psoriatic inflammation [3]. Currently, the interleukin-23 (IL-23)/T helper cell 17 (Th17) axis is recognized as the key driver of psoriasis vulgaris and is the focus of drug development [3]. Specifically, triggers such as trauma, infection (e.g., streptococcal infection), and drugs (e.g., beta blockers, IFN-α, and lithium) stimulate keratinocytes to secrete antimicrobial peptides (ll37, hBD2, hBD3, s100a7–9) [2]. Then, the antibacterial peptides bind to DNAs or RNAs of the damaged cells, which activate the plasma-like dendritic cells that in turn stimulate the myeloid dendritic cells to secrete IL-23, TNF-α, etc. [4]. IL-23-based cytokines polarize Th cells toward Th1, Th17, and Th22 cell subsets, defined by the production of IFN-γ, IL-17A/IL-17F, and IL-22, respectively [5]. In turn, these cytokines result in the excessive proliferation and abnormal differentiation of keratinocytes and prompt keratinocytes to secrete a large number of chemokines (e.g., CXCL1, CXCL3, CXCL5, CXCL8, CCL20) and cytokines (e.g., IL-1, IL-6, IL-36, TNF-α, VEGF) [6]. All of these changes form the pathogenic cycle of keratinocytes and T cells, further aggravating the inflammatory cascades in psoriasis vulgaris [2]. Given this known pathogenesis, many therapies targeting these cells and cytokines have been developed [7]. Anti-TNF-α agents, including infliximab(PASI 75: 80%, week 10), etanercept(PASI 75: 59%, week 12), and adalimumab(PASI 75: 71%, week 16), demonstrate notable efficacy in the treatment of psoriasis firstly [3]. TNF-α is a proinflammatory cytokine produced by dendritic cells, macrophages, and T cells, etc. [8]. It has a broad range of biological effects through the induction of expression of other proinflammatory cytokines and of neutrophil chemoattractants, inducing vascular adhesion molecules facilitating the influx of inflammatory cells, and amplification of the effects of other cytokines, including IL-17 [8]. Then, three anti-IL-17 agents are approved: secukinumab(PASI 75: 82%, week 12), ixekizumab(PASI 75: 90%, week 12), and brodalumab(PASI 75: 86%, week 1) [8]. There are currently four agents that target IL-23 in clinical use for psoriasis: ustekinumab(PASI 75: 64%, week 12), which blocks the common p40 subunit of IL-12 and IL-23, and guselkumab(PASI 90: 73%, week 16), risankizumab(PASI 90: 75%, week 16), and tildrakizumab(PASI 75: 64%, week 12), which target the p19 subunit of IL-23 [8]. Although these monoclonal antibodies have clear effects, no radical treatment that can be used on every patient with psoriasis is available [1]. Therefore, there is an urgent need to further elucidate the molecular mechanism of psoriasis, as well as identify effective new targets with various functions.

Previous research on the role of epigenetics in human diseases mainly focused on DNA and histone modifications, and its biological and pathological functions have been widely proven. Similar to DNA modification, RNA has been demonstrated to contain 172 modifications in the MODOMICS database. This new layer of genetic information is referred to as “epitranscriptomics” [9]. Among the various types, N6-methyladenosine (m^6^A) methylation is the most pervasive and deeply studied mRNA modification [10]. About 20% to 40% of transcripts encoded in mammalian cells are modified by m^6^A methylation, and methylated transcripts often contain multiple m^6^A sites [7]. However, owing to the lack of sensitive detecting technology, there has been only limited exploration of their biological significance. In recent years, with the emergence of antibody-based, high-throughput sequencing, namely, methylated RNA immunoprecipitation with next-generation sequencing (MeRIP-Seq), m^6^A methylation has become a hotspot of scientific research, in which unprecedented progress has been achieved [7]. The biological functions of m^6^A are determined by methyltransferases (“writers”), demethylases (“erasers”), and binding proteins (“readers”). Methyltransferase-like 3 (METTL3) was the first identified m^6^A writer, with methyltransferase-like 14 (METTL14) and Wilms tumor-associated protein (WTAP) subsequently also being identified as methyltransferases [11]. Fat mass and obesity-associated protein (FTO) and α-ketoglutarate-dependent dioxygenase alkB homolog 5 (ALKBH5) were identified as m^6^A demethylases in mRNA [12,13]. The discovery of methyltransferases and demethylases revealed the reversibility and complexity of m^6^A methylation [11]. The identification of different “readers” suggested that m^6^A methylation is likely to affect mRNA fate and have various biological functions in cells [11]. In fact, studies have reported that m^6^A methylation regulates various aspects of mRNA metabolism, including processing, localization, translation, and eventual decay [14]. Furthermore, m^6^A methylation regulates gene expression in different physiological processes, such as cell proliferation, differentiation, and inflammation [14,15,16,17,18,19], which are closely related to the pathogenesis of psoriasis as mentioned above. Studies of aberrant m^6^A in human diseases are rapidly evolving, such as in cancer, obesity, infertility, neuronal disorders, immune diseases, and inflammation [16,19]. However, the regulatory mechanism and physiological significance of m^6^A methylation in psoriasis vulgaris are still poorly understood.

In our previous work, we performed m^6^A transcriptome-wide profiling in psoriatic skin [20]. In this study, we found that m^6^A methylation and METTL3 were both downregulated in psoriatic skin lesions and were negatively correlated with Psoriasis Area and Severity Index (PASI) scores. Inhibiting m^6^A methylation by knocking down *Mettl3* promoted the development of psoriasis and increased its severity in imiquimod-induced (IMQ-induced) psoriasis-like model mice. Our results indicated the critical role of METTL3-mediated m^6^A methylation in the pathogenesis of psoriasis vulgaris.

## 2. Results

### 2.1. Downregulation of m^6^A and METTL3 Level in Psoriatic Skin Lesions

**Figure 1:**
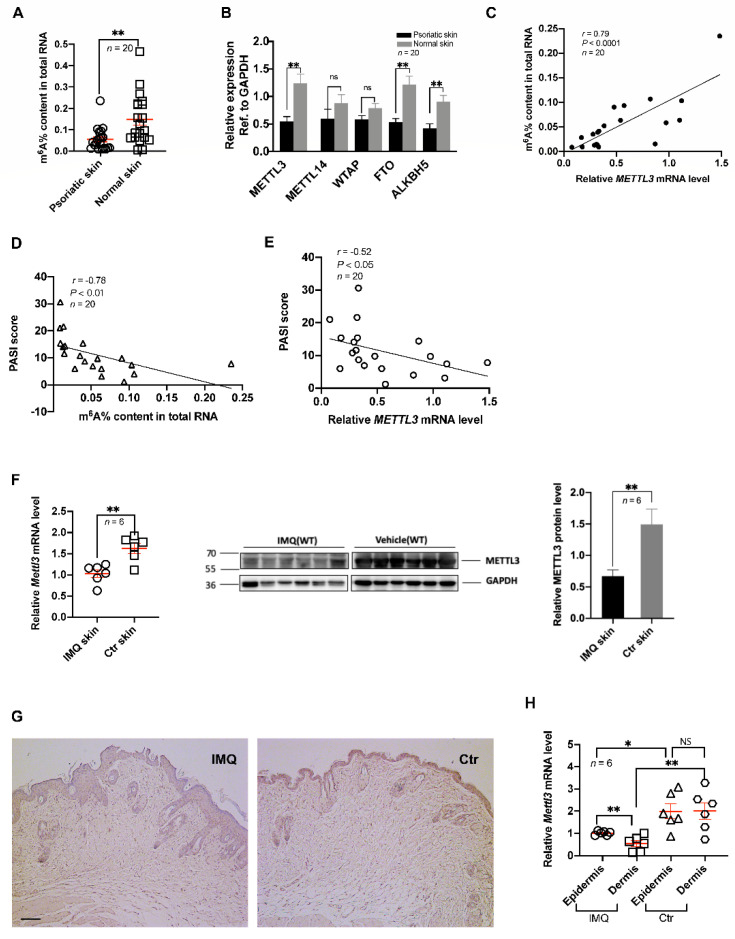
Downregulation of m^6^A and METTL3 levels in psoriatic skin lesions. (**A**) The m^6^A levels of total RNAs in skin samples (*n* = 20) derived from patients with psoriasis vulgaris and healthy controls. (**B**) Expression of METTL3, METTL14, WTAP, FTO, and ALKBH5 in skin samples (*n* = 20) derived from patients with psoriasis vulgaris and healthy controls. (**C**) Correlation of m^6^A levels of total RNAs in human psoriatic skin (*n* = 20) with PASI scores. (**D**,**E**) Correlation of human m^6^A levels of total RNAs (D, *n* = 20) or expression of METTL3 (E, *n* = 20) in human psoriatic skin with PASI scores. (**F**) Expression of *Mettl3* in skin samples from untreated mice (*n* = 6) and IMQ-induced mice (*n* = 6). (**G**) Immunohistochemical analysis was performed on mouse skin treated with vehicle (Ctr, *n* = 6) or IMQ (IMQ, *n* = 6) using anti-METTL3 monoclonal antibodies. Dark brown color indicates *Mettl3* expression. Scale bars: 100 μm. (**H**) Expression of *Mettl3* in the epidermis and dermis of skin samples from untreated controls (*n* = 6) or IMQ-treated mice (*n* = 6). Data represent the mean ± SEM. * *p* < 0.05, ** *p* < 0.01, NS, not significant.Circle, point, triangle and square represent each sample. Data represent the mean ± SEM. Two-tailed Mann–Whitney test (**A**,**B**), two-tailed unpaired Student’s *t* test (**F**,**H**), and Spearman’s r test (**C**,**D**,**E**) were used.

To assess the level of m^6^A methylation in psoriasis vulgaris, we measured the m^6^A level in the total RNA of samples from psoriatic skin lesions and healthy control skin. The results showed that the level of m^6^A methylation in psoriatic skin lesions was significantly lower than that in healthy skin (Figure 1A). This result was consistent with our previous study that the hypomethylated m^6^A peaks/genes (1719/1113) in the skin lesions of psoriasis vulgaris significantly outnumbered the hypermethylated m^6^A peaks/genes (1470/1127) (*p* < 0.0001) [20].

Because reversible m^6^A methylation is regulated by m^6^A methyltransferases and demethylases, we determined the expression levels of some common m^6^A methyltransferases (METTL3, METTL14, WTAP) and demethylases (FTO, ALKBH5) [21]. The results showed that the expression of METTL3, FTO, and ALKBH5 was significantly downregulated in the psoriatic skin lesions compared with that in healthy control skin (Figure 1B). Considering the catalytic ability of these genes and the decreasing m^6^A methylation in psoriasis vulgaris, we speculated that METTL3 plays a more important role in the downregulation of m^6^A, while FTO and ALKBH5 could constitute a feedback mechanism compensating for the declining m^6^A methylation induced by METTL3 in psoriasis vulgaris. Correlation analysis between METTL3 level and m^6^A level showed a significant positive correlation, which agreed with our hypothesis (Figure 1C).

In addition, we established an IMQ-induced psoriasis-like mouse model in accordance with previously reported methods [22]. Consistent with the results from human samples, the expression of *Mettl3* in the IMQ-induced psoriasis-like mice was significantly lower than that in matrix-exposed mice, as assessed by reverse-transcription quantitative PCR (RT-qPCR) and western blotting (Figure 1F). Moreover, to determine the expression of METTL3 in the epidermis and dermis, we conducted immunohistochemical analyses. The results showed that the level of METTL3 decreased in both the epidermis and dermis (Figure 1G), which was consistent with the RT-qPCR analysis of the epidermis and dermis of skin lesions (Figure 1H).

### 2.2. Psoriasis Area and Severity were Associated with Low Levels of m^6^A Methylation and METTL3

PASI, the mostly widely used clinical trial efficacy endpoint, is a single score calculated from the affected body surface area (utilizing a seven-point score for the involvement in each of four anatomical areas: head, upper extremities, trunk, and lower extremities) and from the scores for erythema, induration, and scaling (each scored using a five-point score from 0 to 4); the total score ranges from 0 to 72 [23]. Higher scores usually correspond to more severe disease [23]. To assess the relationship between the extent of m^6^A methylation and PASI scores, we conducted a correlation analysis and found that the level of m^6^A methylation was negatively correlated with the PASI score (Figure 1D). Furthermore, we analyzed the relationship between METTL3 expression and PASI score, the results of which showed that METTL3 expression was also negatively correlated with PASI score (Figure 1E). Based on these results, we assume that METTL3-mediated m^6^A methylation is closely related to the development of psoriasis vulgaris and that with increasing PASI score, m^6^A methylation gradually decreases.

### 2.3. Heterozygous knockout of Mettl3 Accelerated the Development of Psoriasis

**Figure 2:**
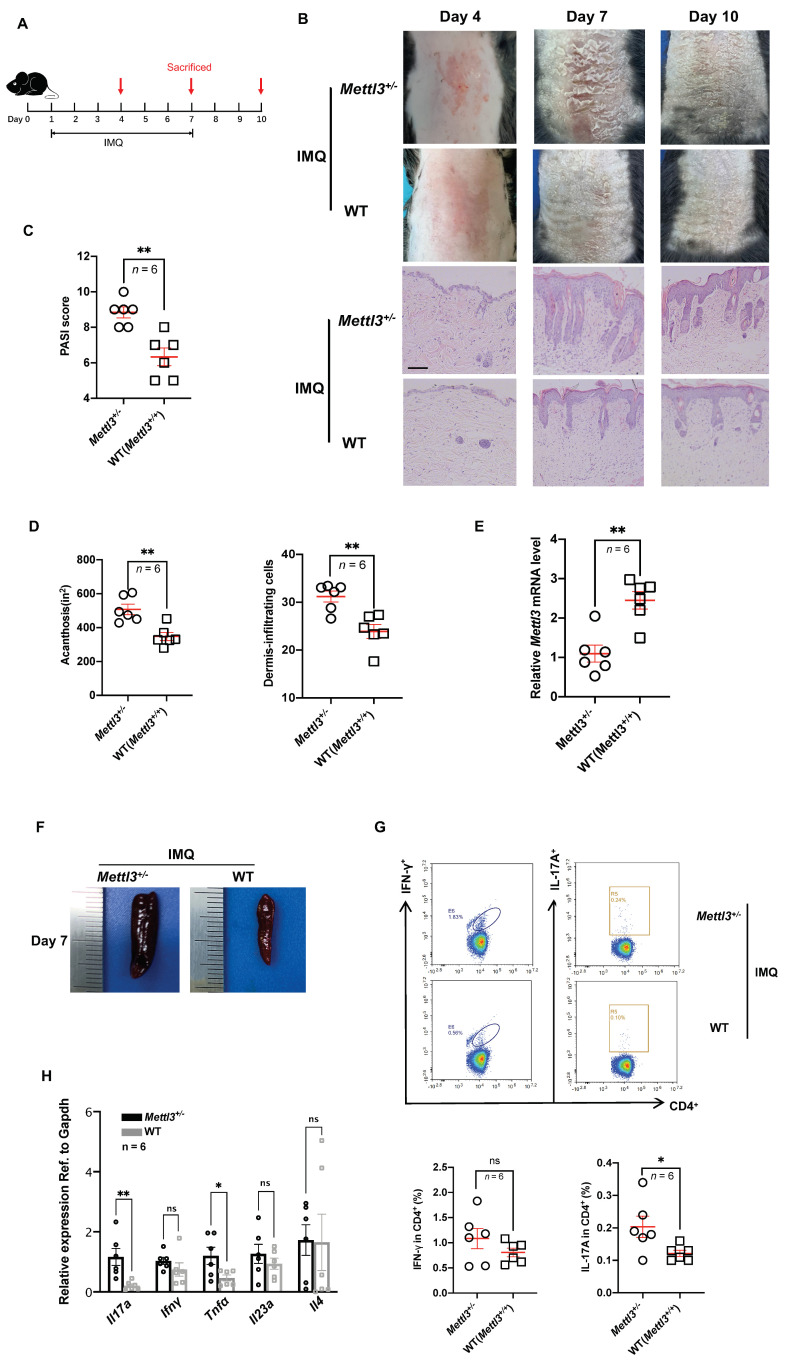
Heterozygous knockout of *Mettl3* accelerated the development of psoriasis. (**A**) Schematic diagram of the experimental plan on IMQ−treated mice (C57BL/6J). Six mice in each group were sacrificed on days 4, 7, and 10 to conduct experiments. (**B**) Phenotypic presentation and H&E staining of lesional skin from *Mettl3^+/−^* and WT (*Mettl3^+/+^*) mice. Scale bars: 100 μm. (**C**) PASI scores of mice in *Mettl3^+/−^* and WT (*Mettl3^+/+^*) groups (*n* = 6). (**D**) Acanthosis and dermal cellular infiltrates were quantified for *Mettl3^+/−^* mice (*n* = 6) or WT (*Mettl3^+/+^*) mice (*n* = 6). For all measurements in D, the median number of specifically stained dermal nucleated cells was counted in six areas in three sections of each sample. (**E**) Expression of *Mettl3* in skin samples from *Mettl3^+/−^* mice (*n* = 6) and WT (*Mettl3^+/+^*) mice (*n* = 6). (**F**) The size of spleens of mice in the *Mettl3^+/−^* and WT (*Mettl3^+/+^*) groups. (**G**) Representative flow cytometric analysis of Th1 and Th17 cells in splenic CD4+ T cells from *Mettl3^+/−^* (*n* = 6) or WT (*Mettl3^+/+^*) (*n* = 6) mice treated with IMQ for 7 days. (**H**) The mRNA levels of *Il17a, Ifnγ, Tnfα, Il23a*, and *Il4* in skin samples from *Mettl3^+/−^* (*n* = 6) or WT (*Mettl3^+/+^*) mice (*n* = 6). Data (C−H) were obtained on the seventh day. Data represent the mean ± SEM. * *p* < 0.05, ** *p* < 0.01. NS, not significant. Circle, point, triangle and square represent each sample. Two-tailed unpaired Student’s *t* test (**C**−**E**,**G**,**H**) was used.

Since *Mettl3* homozygous null (*Mettl3*^−/−^) mice are embryonically lethal [24], *Mettl3* heterozygous knockout (*Mettl3*^+/−^) mice were used for our experiments, and their littermates (*Mettl3*^+/+^) were used as a WT control. Heterozygous knockout of *Mettl3* was confirmed by RT-qPCR and western blotting (Supplemental Figure S1A). Reduced mRNA m^6^A levels in *Mettl3*^+/−^ mice were confirmed by the m^6^A enzyme-linked immunosorbent assay (Supplemental Figure S1B). To study the functional correlation of METTL3 downregulation with the development of psoriasis, we applied 5% imiquimod cream on the back of mice for 7 consecutive days and sacrificed the mice at 4, 7, and 10 days (Figure 2A). Compared with the *Mettl3*^+/+^ group, the *Mettl3*^+/−^ group showed aggravated psoriasis-like clinical and pathological manifestations (Figure 2B), higher PASI scores (Figure 2C), increased acanthosis (an increase in the thickness of the stratum spinosum of the epidermis), and inflammatory cell infiltration in the skin lesions (Figure 2D), which were most pronounced on day 7. We also found that the *Mettl3*^+/−^ group had significantly lower METTL3 levels in skin samples than the *Mettl3*^+/+^ group (Figure 2E). On day 7, at the peak of psoriasis-like changes, we observed that the splenomegaly of mice in the *Mettl3*^+/−^ group was more pronounced than that in the *Mettl3*^+/+^ group (Figure 2F). To detect the infiltration of inflammatory cells in the spleen of mice, we obtained a splenic single-cell suspension and analyzed the proportion of Th1 and Th17 cells among the CD4^+^ T cells in the spleen. The results showed a significant increase in the proportion of Th17 cells among the splenic CD4^+^ T cells of the *Mettl3*^+/−^ group compared with those of the *Mettl3*^+/+^ group (Figure 2G). In addition, compared with the *Mettl3*^+/+^ group, the mRNA expression of *Il17a* and *Tnfα* in skin samples of the *Mettl3*^+/−^ group was significantly higher than that in the *Mettl3*^+/+^ group (Figure 2H). These results demonstrated that inhibiting m^6^A methylation by knocking down *Mettl3* promotes the development of psoriasis and increases its severity in IMQ-induced psoriasis-like mouse models.

### 2.4. m^6^A-seq Identifies Transcripts with Altered Methylation in Psoriasis Vulgaris

**Figure 3:**
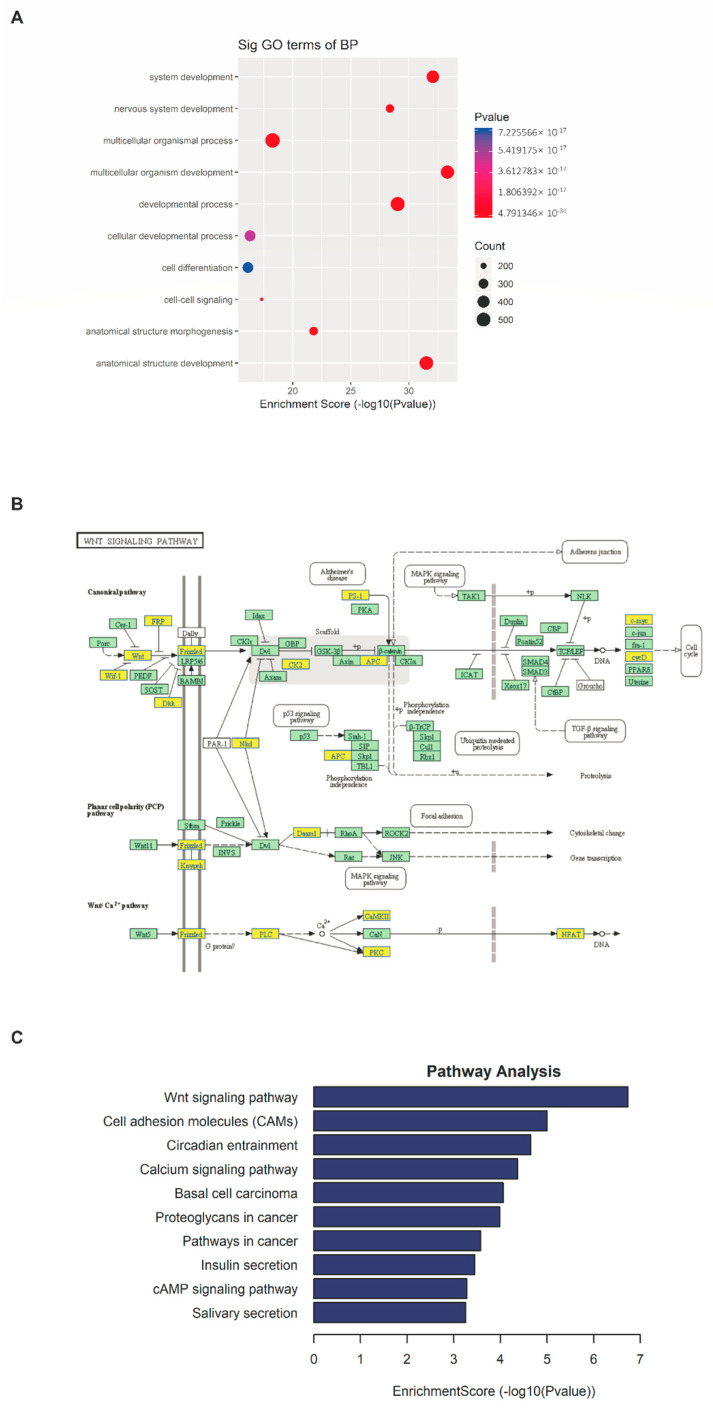
m^6^A−seq identifies transcripts with altered methylation in psoriasis vulgaris. (**A**) The top 10 Gene Ontology terms were significantly enriched for the hypomethylated genes in skin samples derived from patients with psoriasis vulgaris (*n* = 4) and healthy controls (*n* = 4). (**B**) Diagram of the Wnt signaling pathway with genes affected by m^6^A. Hypomethylated genes are marked in yellow and nonmethylated genes are marked in green. The diagram is based on KEGG annotations. (**C**) The top 10 significantly enriched pathways for the hypomethylated genes in skin samples derived from patients with psoriasis vulgaris (*n* = 4) and healthy controls (*n* = 4).

Next, we analyzed the previous m^6^A-seq sequencing data [20]. Through model-based analysis of ChIP-seq (MACS), we identified an average of ~19,000 significant m^6^A peaks in ~17,000 transcripts [20]. The analysis of differentially methylated m^6^A peaks/genes showed that the hypomethylated m^6^A peaks/genes (1719/1113) in the skin lesions of psoriasis vulgaris significantly outnumbered the hypermethylated m^6^A peaks/genes (1470/1127) (*p* < 0.0001) [20]. GO analysis revealed that the transcripts with downregulated m^6^A methylation were enriched for several critical cellular processes, such as cell development, cell differentiation, cell–cell signaling, and cell adhesion (Figure 3A) [20]. KEGG pathway analysis identified the Wnt signaling pathway as being significantly modified by reduced m^6^A (24 genes, *p*-value = 1.86 × 10^−7^) (Figure 3B,C) [20].

## 3. Discussion

Increasing evidence has demonstrated that m^6^A methylation is frequently involved in mediating different physiological processes, such as regulating cell proliferation, stem cell differentiation, immune responses, and tumorigenesis [25,26,27]. However, limited studies have addressed the pathological implications of m^6^A methylation in disease models. In our study, we found that m^6^A methylation was downregulated in psoriatic skin lesions and was negatively correlated with the PASI score, suggesting that m^6^A methylation may be associated with the occurrence and development of psoriasis vulgaris. Moreover, we found that the methyltransferase METTL3 was expressed at a lower level in psoriatic skin lesions and was negatively correlated with the PASI score, indicating its critical role in the downregulation of m^6^A. Furthermore, we functionally demonstrated the essential role of m^6^A methylation in the development of psoriasis-like mouse models. Inhibiting m^6^A methylation by knocking down *Mettl3* promoted the development of psoriasis and increased its severity in imiquimod-induced psoriasis-like model mice. These results demonstrated that the downregulated METTL3-mediated low level of m^6^A contributes to the progression of psoriasis vulgaris.

The effect of m^6^A methylation on cell proliferation and differentiation is one of the critical mechanisms involved in tumor progression [28]. However, the outcome of m^6^A methylation on cell proliferation and differentiation differs in different conditions or diseases. Several studies have reported that m^6^A methylation can promote cell proliferation or inhibit cell differentiation. For example, in acute myeloid leukemia (AML), overexpressed METTL3 promotes cell proliferation and inhibits cell differentiation by modifying c-MYC, BCL-2, and PTEN with m^6^A [27]. WTAP, another methyltransferase, also promotes cell proliferation and inhibits the cell differentiation of AML [29]. In glioma stem-like cells, METTL3 and METTL14 were also shown to suppress cell differentiation, while WTAP and ALKBH5 promote cell proliferation, self-renewal, and tumorigenicity [13,30,31,32]. Conversely, other studies revealed that m^6^A methylation inhibited cell proliferation or promoted cell differentiation. For example, a study on renal cell carcinoma showed that the depletion of METTL3 promoted cell proliferation, cell growth, and colony formation [33]. In addition, Huang et al. [34] reported that the ester form of meclofenamic acid increased the m^6^A level and resulted in the arrest of the G1/S transition and a decrease in spermatogonial proliferation. In glioblastoma, elevated ALKBH5 enhanced cell self-renewal, proliferation, and tumorigenicity by the demethylation of m6A [13]. In cervical cancer, decreased m^6^A is closely correlated with differentiation, and the reduction of m^6^A promotes cell proliferation and tumor development [35]. Geula et al. [24]. reported that m^6^A mRNA methylation facilitates the resolution of naive pluripotency toward differentiation. Other than these cancer cells, m^6^A methylation is also involved in regulating the proliferation and apoptosis of epithelial cells [36,37]. In human lens epithelial cells, inhibition of the m^6^A level by knocking down METTL3 promoted the proliferation and repressed the apoptosis of these cells induced by high glucose [36]. Moreover, in human bronchial epithelial cells, METTL3 heterozygous knockout led to an inversion in the inordinate human bronchial epithelial cell proliferation and apoptosis in arsenite-transformed cells [37]. m^6^A has also been shown to control T-cell homeostasis and differentiation [38,39]. These different functions of m^6^A methylation may be related to different enzymes, tissues, and cells [40]. In our study, reduced m^6^A increased acanthosis and the proportion of Th17 cells in a psoriasis-like mouse model, suggesting that reduced m^6^A methylation might promote the proliferation of keratinocytes and the differentiation of naïve T cells to Th17 cells in psoriasis vulgaris. Because the uncontrolled proliferation of keratinocytes and dysfunctional differentiation of T cells are known to be hallmarks of psoriasis vulgaris [2], we assumed that m^6^A methylation might serve as an important regulator in the pathogenesis of this disease.

m^6^A methylation has also been implicated in immune and inflammation regulation [18]. A study on human dental pulp inflammation showed that METTL3 heterozygous knockout inhibited inflammatory cytokine production by affecting the alternative splicing of MyD88 [18]. In addition, a study on osteoarthritis showed that the silencing of METTL3 suppressed IL-1β-induced inflammatory cytokines and the NF-κB signaling pathway in chondrocytes [41]. Conversely, a study on human rheumatoid arthritis showed that the overexpression of METTL3 significantly inhibited the inflammatory response induced by lipopolysaccharide in macrophages, including decreasing the production of inflammation-associated cytokines (IL-6 and TNF-α) and the proliferation of macrophages [42]. Li et al. [38]. reported that deleting *Mettl3* in mouse T cells led to the development of chronic inflammation of the intestine in mice. In addition, Yu et al. [43]. reported that heterozygous knockout of the m^6^A reader YTHDF2 significantly increased the lipopolysaccharide-induced inflammatory cytokines (IL-6, TNF-α, IL-1β, and IL-12) and the phosphorylation of p65, p38, and ERK1/2 in NF-κB and MAPK signaling. In our study, reduced m^6^A promoted inflammatory cell infiltration in the skin lesions and increased the expression levels of inflammatory cytokines closely related to psoriasis vulgaris, such as *Il17a* and *Tnfα*. These results suggested that m^6^A methylation is involved in regulating the inflammatory response of psoriatic lesions. Consistent with our results, Li et al. [44]. found that high levels of m^6^A induced by overexpressed WTAP reduced T-cell infiltration and inhibited tumor immunity in gastric cancer. They thought that these results were because of the control of IFN mRNA expression by m^6^A [44]. In addition, a study reported that D2-like dopamine receptors, which play an important role in modulating T-lymphocyte development and function, are strongly influenced by m^6^A methylation [45].

According to our previous m^6^A-seq data, the hypomethylated transcripts in psoriasis were mainly associated with the Wnt signaling pathway (24 genes, *p*-value = 1.86e-7) [20]. Several studies have demonstrated that multiple transcripts in the Wnt signaling pathway were modified by m^6^A and that this modification regulates their mRNA and protein levels, and then participates in the pathogenesis of diseases [46,47,48,49]. For example, m^6^A suppression promoted gastric cancer cell proliferation and invasiveness through activating Wnt and PI3K-Akt signaling [48]. In addition, the Wnt signaling pathway is involved in many cellular functions that are essential for normal organ development, including cell proliferation, survival, self-renewal, and differentiation [50]. In psoriasis vulgaris, the Wnt signaling pathway is involved in regulating the inflammatory cascade, and the differentiation, proliferation, and apoptosis of keratinocytes [51,52,53], which are all among the most critical factors in the pathogenesis of psoriasis vulgaris [2]. These findings indicate that m^6^A methylation is an important RNA epigenetic marker that is involved in regulating the expression of genes with important biological functions in psoriasis vulgaris, such as cell differentiation, cell differentiation, and inflammation. We hypothesize that the reduction of m^6^A methylation exacerbates the development of psoriasis by regulating the Wnt signaling pathway. However, this hypothesis and the specific molecular mechanism involved require further study to verify.

## 4. Materials and Methods

### 4.1. Patient Samples

A total of 20 patients, who were diagnosed with psoriasis vulgaris based on clinical manifestations and pathological examination, were recruited from the dermatology clinic of Peking Union Medical College Hospital. Psoriasis disease activity was assessed using the PASI score. Information on the patients is shown in Supplemental Table S1. A total of 20 sex- and age-matched healthy controls were also recruited from the Department of Plastic Surgery at Peking Union Medical College Hospital. All human studies were approved by the ethics committee of Peking Union Medical College Hospital. Written informed consent was obtained from all subjects.

### 4.2. Mice

The C57BL/6J mice were purchased from Vital River Laboratory Animal Technology Co., Ltd. (Beijing, China). *Mettl3*^+/−^ and *Mettl3*^+/+^ mice were generated by GemPharmatech Co., Ltd. (Jiangsu, China). CRISPR/Cas9 technology was used to modify the *Mettl3* gene. The process can be described in brief as follows: sgRNA was transcribed in vitro. Cas9 and sgRNA were microinjected into the fertilized eggs of C57BL/6J mice. These fertilized eggs were then transplanted to obtain positive F0 mice, which were confirmed by PCR and sequencing. A stable F1 generation mouse model was obtained by mating positive F0 generation mice with C57BL/6J mice. Because *Mettl3* homozygous null (*Mettl3*^−/−^) mice are embryonically lethal [24], *Mettl3* heterozygous knockout (*Mettl3*^+/−^) mice were used for our experiments and their littermates (*Mettl3*^+/+^) were used as a wild type (WT) control. Female mice at 6–8 weeks of age were used for all experiments. All mice were maintained in specific pathogen-free conditions. The animal care and experimental procedures were approved by the Animal Care Committee of Peking Union Medical College Hospital. All animal experiments followed the ARRIVE guidelines.

### 4.3. IMQ-Induced Psoriasis-like Mouse Model

Female *Mettl3*^+/−^ and *Mettl3*^+/+^ mice on a C57BL/6J background (6–8 weeks of age) were kept under controlled conditions. The mice were treated with a daily topical dose of 62.5 mg of IMQ cream (5%) (Aldara; 3M Pharmaceuticals, St. Paul, MN, USA) on the shaved back for 7 consecutive days and then sacrificed for the subsequent experiments. Control mice were treated with the same dose of vehicle cream.

### 4.4. Scoring the Severity of Skin Inflammation in Psoriasis-like Mouse Model

An objective scoring system was developed to score the severity of back skin inflammation in the IMQ-induced psoriasis-like mouse model, which was based on the clinical PASI score for assessing patients with psoriasis. Erythema, thickening, and scaling were scored independently on a scale from 0 to 4 as follows: 0, none; 1, slight; 2, moderate; 3, marked; and 4, very marked. The total score is the sum of the three index scores (scores 0–12).

### 4.5. RNA m^6^A Quantification

Total RNA from skin samples was extracted using TRIzol reagent (Invitrogen, Carlsbad, CA, USA) and a NanoDrop spectrophotometer (Genova Nano; Jenway, Stone, UK) was used for RNA quality control. The m^6^A content in the total RNAs was detected using the m^6^A RNA methylation quantification kit (Abcam, Cambridge, MA, USA). Briefly, 200 ng of RNA from skin samples was added to the designated wells. Capture antibody solution and detection antibody solution were then added to assay the wells separately at a suitable diluted concentration, following the manufacturer’s instructions. The m^6^A levels were quantified colorimetrically by reading the absorbance of each well at a wavelength of 450 nm, and then calculations were performed based on the standard curve.

### 4.6. Flow Cytometry

The proportion of Th1 and Th17 cells among the splenic CD4^+^ T cells of IMQ-induced mice was determined by flow cytometry. In brief, single-cell suspensions were obtained using filter grinding and fluorescent-activated cell sorting lysing solution. Then, the cells were stimulated for 5 h with phorbol myristate acetate (Sigma, St. Louis, MO, USA), ionomycin (Sigma), and protein transport inhibitor (Sigma). Then, the cells were incubated with FITC anti-mouse CD3 (Biolegend, San Diego, CA, USA) and APC anti-mouse CD4 (Biolegend) for 30 min on ice in the dark. After fixation with 2% formaldehyde for 20 min and permeabilization for 30 min, the cells were stained with PE anti-mouse IL-17A (Biolegend) and PerCp anti-mouse IFN-γ (Biolegend) on ice for an additional 30 min in the dark. Cells were analyzed using ACEA NovoCyte (ACEA Biosciences, San Diego, CA, USA) and data were analyzed using the FlowJo software (Tree Star, San Carlos, CA, USA).

### 4.7. RT-qPCR

Total RNA from skin samples was extracted using TRIzol reagent (Invitrogen). The NanoDrop spectrophotometer (Genova Nano) was used for RNA quality control. For the separation of the dermis and epidermis, skin samples were incubated with 1 mol/L NaCl solution at 4 °C for 24 h. mRNA was reverse-transcribed with the PrimeScript RT Reagent Kit with gDNA Eraser (TaKaRa, Otsu, Japan). PCR was conducted with the SYBR PrimeScript Reverse-Transcription PCR kit (TaKaRa). Amplification was implemented using the ABI 7500 Fast Real-Time PCR System (Applied Biosystems, Foster City, CA, USA). The relative gene expression level was calculated using 2–(gene–GAPDH), involving normalization to the internal control gene GAPDH. The fold change of gene expression was calculated using 2–(ΔCt experimental group–ΔCt control group), with normalization to the control group. Primers for *METTL3, TNF-α, IL17-a, IL-4, IL23-a, IFN-γ*, and *GAPDH* were obtained from Majorbio (Shanghai, China) and primer sequences are presented in Supplemental Table S2.

### 4.8. Western Blotting

Skin samples were extracted using the Total Protein Extraction Kit for Skin Tissue (Invent Biotechnologies, Plymouth, MN, USA). Protein concentration was measured using the BCA Protein Assay Kit (Beyotime, Shanghai, China). Proteins were subjected to analysis using 10% SDS-PAGE gels under denaturing conditions and then transferred onto a PVDF membrane (Millipore, Billerica, MA, USA). Anti-mouse METTL3 Ab (Proteintech, Rosemont, IL, USA) or anti-human METTL3 Ab (Affinity, Cincinnati, OH, USA) was used as the primary antibody, and goat anti-rabbit/mouse IgG H&L (HRP) Ab (Affinity) was used as the secondary antibody. Membranes were developed with a detection solution (Beyotime) and the protein side of each membrane was exposed to an image analysis system (Tanon, Shanghai, China). The quantification of METTL3 was normalized to GAPDH by densitometry.

### 4.9. Histological Analysis and Immunohistochemistry

Histological analysis and immunohistochemistry were conducted as previously reported [54,55]. Human and mouse skin tissues were fixed in formalin and embedded in paraffin. Sections (6 μm) were stained with hematoxylin and eosin (H&E) and stored at room temperature. Acanthosis (epidermal hyperplasia) and the number of dermis-infiltrating cells were assessed as histological features. Briefly, for measuring acanthosis, the epidermal area was outlined and its pixel size was measured using the Lasso tool in Preview. The relative area of the epidermis was calculated using the following formula: area = pixels/(horizontal resolution × vertical resolution). For counting dermis-infiltrating cells, six areas (of 9 cm^2^ each) in three sections of each sample were randomly selected, in which the number of infiltrating cells was counted. For immunohistochemical analysis, METTL3 expression was evaluated in skin sections using rabbit anti-mouse METTL3 Ab (Proteintech), following the manufacturer’s instructions.

### 4.10. Statistical Analyses

Statistical analysis was performed using GraphPad Prism 8.0 software. Data are presented as mean ± SEM. Different statistical methods were used according to the data type. Student’s *t*-test (bilateral) was used for the analysis of continuous variables between the two groups. When the data were not normally distributed or displayed unequal variances between two groups, we used the two-tailed Mann–Whitney test for statistical analysis. Pearson’s correlation was used to analyze the correlation. When *p* < 0.05, the difference was considered statistically significant. * *p* < 0.05, ** *p* < 0.01, *** *p* < 0.001.

## 5. Conclusions

In conclusion, psoriasis vulgaris is triggered by environmental and genetic stimuli, between which lie epigenetic factors. Extensive epidemiological and molecular analyses have provided evidence for the involvement of epigenetics in psoriasis vulgaris, especially DNA methylation and histone modification. However, the biological significance of RNA methylation in psoriasis vulgaris remains to be explored in depth. In our study, we found that the reduction of METTL3-mediated m^6^A methylation exacerbated the development of psoriasis vulgaris in an IMQ-induced psoriasis-like mouse model. Inhibiting m^6^A methylation by knocking down *Mettl3* increased acanthosis, the proportion of Th17 cells, and inflammatory cell infiltration in the psoriasis-like mouse model. In addition, the reduced m^6^A methylation might accelerate the development of psoriasis by regulating the Wnt signaling pathway. Our study demonstrated that METTL3-mediated m^6^A methylation participates in processes that are critical for the development of psoriasis vulgaris. In future work, the specific molecular mechanism by which METTL3-mediated RNA m^6^A methylation regulates the occurrence and development of psoriasis vulgaris should be further studied.

## Data Availability

The sequencing datasets are available in the NCBI repository, https://www.ncbi.nlm.nih.gov/geo/query/acc.cgi?acc=GSE155702(accessed on 1 December 2020). Other datasets used and/or analysed during the current study are available from the corresponding author on reasonable request.

## Supplementary Materials

The following supporting information can be downloaded at: https://www.mdpi.com/article/10.3390/ijms232012672/s1.

## References

[1] Greb J.E., Goldminz A.M., Elder J.T., Lebwohl M.G., Gladman D.D., Wu J.J., Mehta N.N., Finlay A.Y., Gottlieb A.B. (2016). Psoriasis. Nat. Rev. Dis. Prim..

[2] Boehncke W.H., Schön M.P. (2015). Psoriasis. Lancet.

[3] Hugh J.M., Weinberg J.M. (2018). Update on the pathophysiology of psoriasis. Cutis.

[4] Becher B., Pantelyushin S. (2012). Hiding under the skin: Interleukin-17-producing γδ T cells go under the skin?. Nat. Med..

[5] Gutcher I., Becher B. (2007). APC-derived cytokines and T cell polarization in autoimmune inflammation. J. Clin. Invest..

[6] Kagami S., Rizzo H.L., Lee J.J., Koguchi Y., Blauvelt A. (2010). Circulating Th17, Th22, and Th1 cells are increased in psoriasis. J. Invest. Dermatol..

[7] Dominissini D., Moshitch-Moshkovitz S., Schwartz S., Salmon-Divon M., Ungar L., Osenberg S., Cesarkas K., Jacob-Hirsch J., Amariglio N., Kupiec M. (2012). Topology of the human and mouse m6A RNA methylomes revealed by m6A-seq. Nature.

[8] Griffiths C.E.M., Armstrong A.W., Gudjonsson J.E., Barker J. (2021). Psoriasis. Lancet.

[9] Schwartz S. (2016). Cracking the epitranscriptome. RNA.

[10] Frye M., Harada B.T., Behm M., He C. (2018). RNA modifications modulate gene expression during development. Science.

[11] Meyer K.D., Jaffrey S.R. (2017). Rethinking m(6)A Readers, Writers, and Erasers. Annu. Rev. Cell Dev. Biol..

[12] Jia G., Fu Y., Zhao X., Dai Q., Zheng G., Yang Y., Yi C., Lindahl T., Pan T., Yang Y.G. (2011). N6-methyladenosine in nuclear RNA is a major substrate of the obesity-associated FTO. Nat. Chem. Biol..

[13] Zhang S., Zhao B.S., Zhou A., Lin K., Zheng S., Lu Z., Chen Y., Sulman E.P., Xie K., Bögler O. (2017). m(6)A Demethylase ALKBH5 Maintains Tumorigenicity of Glioblastoma Stem-like Cells by Sustaining FOXM1 Expression and Cell Proliferation Program. Cancer Cell.

[14] Roundtree I.A., Evans M.E., Pan T., He C. (2017). Dynamic RNA Modifications in Gene Expression Regulation. Cell.

[15] Li F., Zhang C., Zhang G. (2019). m6A RNA Methylation Controls Proliferation of Human Glioma Cells by Influencing Cell Apoptosis. Cytogenet. Genome Res..

[16] Maity A., Das B. (2016). N6-methyladenosine modification in mRNA: Machinery, function and implications for health and diseases. FEBS J..

[17] Wang Y.N., Yu C.Y., Jin H.Z. (2020). RNA N(6)-Methyladenosine Modifications and the Immune Response. J. Immunol. Res..

[18] Feng Z., Li Q., Meng R., Yi B., Xu Q. (2018). METTL3 regulates alternative splicing of MyD88 upon the lipopolysaccharide-induced inflammatory response in human dental pulp cells. J. Cell Mol. Med..

[19] Lv X., Liu X., Zhao M., Wu H., Zhang W., Lu Q., Chen X. (2021). RNA Methylation in Systemic Lupus Erythematosus. Front. Cell Dev. Biol..

[20] Wang Y.N., Jin H.Z. (2020). Transcriptome-Wide m(6)A Methylation in Skin Lesions From Patients With Psoriasis Vulgaris. Front. Cell Dev. Biol..

[21] Zhao B.S., Roundtree I.A., He C. (2017). Post-transcriptional gene regulation by mRNA modifications. Nat. Rev. Mol. Cell Biol..

[22] van der Fits L., Mourits S., Voerman J.S., Kant M., Boon L., Laman J.D., Cornelissen F., Mus A.M., Florencia E., Prens E.P. (2009). Imiquimod-induced psoriasis-like skin inflammation in mice is mediated via the IL-23/IL-17 axis. J. Immunol..

[23] Mattei P.L., Corey K.C., Kimball A.B. (2014). Psoriasis Area Severity Index (PASI) and the Dermatology Life Quality Index (DLQI): The correlation between disease severity and psychological burden in patients treated with biological therapies. J. Eur. Acad. Dermatol. Venereol..

[24] Geula S., Moshitch-Moshkovitz S., Dominissini D., Mansour A.A., Kol N., Salmon-Divon M., Hershkovitz V., Peer E., Mor N., Manor Y.S. (2015). Stem cells. m6A mRNA methylation facilitates resolution of naïve pluripotency toward differentiation. Science.

[25] Batista P.J., Molinie B., Wang J., Qu K., Zhang J., Li L., Bouley D.M., Lujan E., Haddad B., Daneshvar K. (2014). m(6)A RNA modification controls cell fate transition in mammalian embryonic stem cells. Cell Stem. Cell.

[26] Liu J., Eckert M.A., Harada B.T., Liu S.M., Lu Z., Yu K., Tienda S.M., Chryplewicz A., Zhu A.C., Yang Y. (2018). m(6)A mRNA methylation regulates AKT activity to promote the proliferation and tumorigenicity of endometrial cancer. Nat. Cell Biol..

[27] Vu L.P., Pickering B.F., Cheng Y., Zaccara S., Nguyen D., Minuesa G., Chou T., Chow A., Saletore Y., MacKay M. (2017). The N(6)-methyladenosine (m(6)A)-forming enzyme METTL3 controls myeloid differentiation of normal hematopoietic and leukemia cells. Nat. Med..

[28] Liu Z.X., Li L.M., Sun H.L., Liu S.M. (2018). Link Between m6A Modification and Cancers. Front. Bioeng. Biotechnol..

[29] Bansal H., Yihua Q., Iyer S.P., Ganapathy S., Proia D.A., Penalva L.O., Uren P.J., Suresh U., Carew J.S., Karnad A.B. (2014). WTAP is a novel oncogenic protein in acute myeloid leukemia. Leukemia.

[30] Jin D.I., Lee S.W., Han M.E., Kim H.J., Seo S.A., Hur G.Y., Jung S., Kim B.S., Oh S.O. (2012). Expression and roles of Wilms’ tumor 1-associating protein in glioblastoma. Cancer Sci..

[31] Cui Q., Shi H., Ye P., Li L., Qu Q., Sun G., Sun G., Lu Z., Huang Y., Yang C.G. (2017). m(6)A RNA Methylation Regulates the Self-Renewal and Tumorigenesis of Glioblastoma Stem Cells. Cell Rep..

[32] Visvanathan A., Patil V., Arora A., Hegde A.S., Arivazhagan A., Santosh V., Somasundaram K. (2018). Essential role of METTL3-mediated m(6)A modification in glioma stem-like cells maintenance and radioresistance. Oncogene.

[33] Li X., Tang J., Huang W., Wang F., Li P., Qin C., Qin Z., Zou Q., Wei J., Hua L. (2017). The M6A methyltransferase METTL3: Acting as a tumor suppressor in renal cell carcinoma. Oncotarget.

[34] Huang T., Guo J., Lv Y., Zheng Y., Feng T., Gao Q., Zeng W. (2019). Meclofenamic acid represses spermatogonial proliferation through modulating m(6)A RNA modification. J. Anim. Sci. Biotechnol..

[35] Wang X., Li Z., Kong B., Song C., Cong J., Hou J., Wang S. (2017). Reduced m(6)A mRNA methylation is correlated with the progression of human cervical cancer. Oncotarget.

[36] Yang J., Liu J., Zhao S., Tian F. (2020). N(6)-Methyladenosine METTL3 Modulates the Proliferation and Apoptosis of Lens Epithelial Cells in Diabetic Cataract. Mol. Ther. Nucleic Acids.

[37] Gu S., Sun D., Dai H., Zhang Z. (2018). N(6)-methyladenosine mediates the cellular proliferation and apoptosis via microRNAs in arsenite-transformed cells. Toxicol. Lett..

[38] Li H.B., Tong J., Zhu S., Batista P.J., Duffy E.E., Zhao J., Bailis W., Cao G., Kroehling L., Chen Y. (2017). m(6)A mRNA methylation controls T cell homeostasis by targeting the IL-7/STAT5/SOCS pathways. Nature.

[39] Yang J., Wang H., Zhang W. (2019). Regulation of Virus Replication and T Cell Homeostasis by N(6)-Methyladenosine. Virol. Sin..

[40] Gilbert W.V., Bell T.A., Schaening C. (2016). Messenger RNA modifications: Form, distribution, and function. Science.

[41] Liu Q., Li M., Jiang L., Jiang R., Fu B. (2019). METTL3 promotes experimental osteoarthritis development by regulating inflammatory response and apoptosis in chondrocyte. Biochem. Biophys. Res. Commun..

[42] Wang J., Yan S., Lu H., Wang S., Xu D. (2019). METTL3 Attenuates LPS-Induced Inflammatory Response in Macrophages via NF-κB Signaling Pathway. Mediat. Inflamm..

[43] Yu R., Li Q., Feng Z., Cai L., Xu Q. (2019). m6A Reader YTHDF2 Regulates LPS-Induced Inflammatory Response. Int. J. Mol. Sci..

[44] Li H., Su Q., Li B., Lan L., Wang C., Li W., Wang G., Chen W., He Y., Zhang C. (2020). High expression of WTAP leads to poor prognosis of gastric cancer by influencing tumour-associated T lymphocyte infiltration. J. Cell Mol. Med..

[45] Hess M.E., Hess S., Meyer K.D., Verhagen L.A., Koch L., Brönneke H.S., Dietrich M.O., Jordan S.D., Saletore Y., Elemento O. (2013). The fat mass and obesity associated gene (Fto) regulates activity of the dopaminergic midbrain circuitry. Nat. Neurosci..

[46] Zhang S.Y., Zhang S.W., Fan X.N., Meng J., Chen Y., Gao S.J., Huang Y. (2019). Global analysis of N6-methyladenosine functions and its disease association using deep learning and network-based methods. PLoS Comput. Biol..

[47] Liu L., Wang J., Sun G., Wu Q., Ma J., Zhang X., Huang N., Bian Z., Gu S., Xu M. (2019). m(6)A mRNA methylation regulates CTNNB1 to promote the proliferation of hepatoblastoma. Mol. Cancer.

[48] Zhang C., Zhang M., Ge S., Huang W., Lin X., Gao J., Gong J., Shen L. (2019). Reduced m6A modification predicts malignant phenotypes and augmented Wnt/PI3K-Akt signaling in gastric cancer. Cancer Med..

[49] Fukumoto T., Zhu H., Nacarelli T., Karakashev S., Fatkhutdinov N., Wu S., Liu P., Kossenkov A.V., Showe L.C., Jean S. (2019). N(6)-Methylation of Adenosine of FZD10 mRNA Contributes to PARP Inhibitor Resistance. Cancer Res..

[50] Duchartre Y., Kim Y.M., Kahn M. (2016). The Wnt signaling pathway in cancer. Crit. Rev. Oncol. Hematol..

[51] Tian R., Li Y., Yao X. (2016). PGRN Suppresses Inflammation and Promotes Autophagy in Keratinocytes Through the Wnt/β-Catenin Signaling Pathway. Inflammation.

[52] Zhang Y., Tu C., Zhang D., Zheng Y., Peng Z., Feng Y., Xiao S., Li Z. (2015). Wnt/β-Catenin and Wnt5a/Ca Pathways Regulate Proliferation and Apoptosis of Keratinocytes in Psoriasis Lesions. Cell Physiol. Biochem..

[53] Wang W., Yu X., Wu C., Jin H. (2017). IL-36γ inhibits differentiation and induces inflammation of keratinocyte via Wnt signaling pathway in psoriasis. Int. J. Med. Sci..

[54] Yan S., Xu Z., Lou F., Zhang L., Ke F., Bai J., Liu Z., Liu J., Wang H., Zhu H. (2015). NF-κB-induced microRNA-31 promotes epidermal hyperplasia by repressing protein phosphatase 6 in psoriasis. Nat. Commun..

[55] Wu R., Zeng J., Yuan J., Deng X., Huang Y., Chen L., Zhang P., Feng H., Liu Z., Wang Z. (2018). MicroRNA-210 overexpression promotes psoriasis-like inflammation by inducing Th1 and Th17 cell differentiation. J. Clin. Invest..

