# Supplementary material for: Reduction of Methyltransferase-like 3-Mediated RNA N6-Methyladenosine Exacerbates the Development of Psoriasis Vulgaris in Imiquimod-Induced Psoriasis-like Mouse Model
Source: Int J Mol Sci. 2022 Oct 21;23(20):12672. doi: 10.3390/ijms232012672 (PMC9603933; doi:10.3390/ijms232012672)
Supplement: Supplementary file 1 [file ijms-23-12672-s001.zip › ijms-1880722-supplementary.pdf]

A

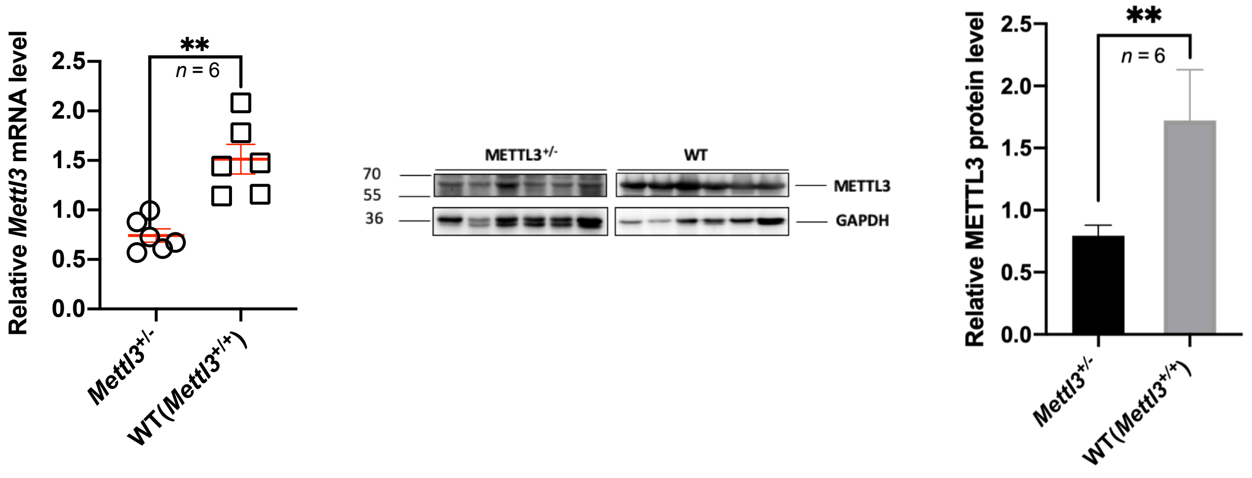

B

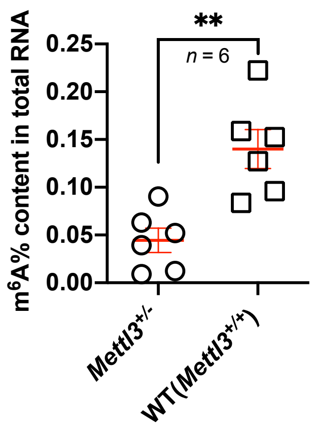

Supplemental Figure S1. Confirmation of *Mettl3* heterozygous knockout mice. (A) Expression of *Mettl3* in skin samples from *Mettl3*$^{+/-}$ mice (n = 6) and WT (*Mettl3*$^{+/+}$) mice (n = 6). (B) The m^{6}A levels of total RNAs in skin samples (n = 20) from *Mettl3*$^{+/-}$ mice (n = 6) and WT (*Mettl3*$^{+/+}$) mice (n = 6). Data represent the mean ± SEM. ***P < 0.001. Two-tailed unpaired Student's t-test (A and B) was used.

Supplemental Table S1. Information for patients with psoriasis vulgaris

Sample ID	Age	Gender	PASI score
1	M	25	8.7
2	M	28	14.1
3	M	42	7.8
4	M	30	10.8
5	M	33	21
6	M	32	3.1
7	M	58	30.6
8	F	28	4
9	F	26	11.6
10	M	31	9.8
11	F	60	7.4
12	F	28	9.7
13	F	54	15.4
14	F	29	6
15	M	33	21.6
16	F	29	15.4
17	F	64	1.2
18	M	52	14.4
19	F	23	6
20	F	64	6.9

Supplemental Table S2. Quantitative PCR primer sequences.

Human-*METTL3*-F	5'-TTGTCTCCAACCTTCCGTAGT-3'
Human-*METTL3*-R	5'-CCAGATCAGAGAGGTGGTGTAG-3'
Human-*METTL14*-F	5'-GAGTGTGTTTACGAAAATGGGGT-3'
Human-*METTL14*-R	5'-CCGTCTGTGCTACGCTTCA-3'
Human-*WTAP*-F	5'-CTTCCCAAGAAGGTTCGATTGA-3'
Human-*WTAP*-R	5'-TCAGACTCTCTTAGGCCAGTTAC-3'
Human-*FTO*-F	5'-ACTTGGCTCCCTTATCTGACC-3'
Human-*FTO*-R	5'-TGTGCAGTGTGAGAAAGGCTT-3'
Human-*ALKBH5*-F	5'-CGGCGAAGGCTACACTTACG-3'
Human-*ALKBH5*-R	5'-CCACCAGCTTTTGGATCACCA-3'
Human-*GAPDH*-F	5'-TCAACGACCACTTTGTCAAGCTCA-3'
Human-*GAPDH*-R	5'-GCTGGTGGTCCAGGGGTCTTACT-3'
Mouse-*Mettl3*-F	5'-CACGCTGCCTCCGATGTTGAT-3'
Mouse-*Mettl3*-R	5'-CTGACCTTCTTGCTCTGCTGTTCTT-3'
Mouse-*Il17a*-F	5'-TCTTTAACTCCCTTGGCGCA-3'
Mouse-*Il17a*-R	5'-TCAGGGTCTTCATTGCGGTG-3'
Mouse-*Il23a*-F	5'-GCTGTGCCTAGGAGTAGCAG-3'
Mouse-*Il23a*-R	5'-CACTGGATACGGGGCACATT-3'
Mouse-*Il4*-F	5'-GATGGATGTGCCAAACGTCC-3'
Mouse-*Il4*-R	5'-CTTGGAAGCCCTACAGACGA-3'
Mouse-*Ifnγ*-F	5'-AACTCAAGTGGCATAGATGTGGAAGA-3'
Mouse-*Ifnγ*-R	5'-AATGACGCTTATGTTGTTGCTGATGG-3'
Mouse-*Tnfα*-F	5'-AGTGGTCAGGTTGCCTCTGTCTC-3'
Mouse-*Tnfα*-R	5'-TCTGGAAAGGTCTGAAGGTAGGAAGG-3'
Mouse-*Gapdh*-F	5'-AGAAGGTGGTGAAGCAGGCATCT-3'
Mouse-*Gapdh*-R	5'-CGGCATCGAAGGTGGAAGAGTG-3'
